# Outcome of pyometra in female dogs and predictors of peritonitis and prolonged postoperative hospitalization in surgically treated cases

**DOI:** 10.1186/1746-6148-10-6

**Published:** 2014-01-07

**Authors:** Supranee Jitpean, Bodil Ström-Holst, Ulf Emanuelson, Odd V Höglund, Ann Pettersson, Caroline Alneryd-Bull, Ragnvi Hagman

**Affiliations:** 1 Department of Clinical Sciences, Faculty of Veterinary Medicine and Animal Science, Swedish University of Agricultural Sciences, Box 7054, SE-750 07 Uppsala, Sweden; 2 Djursjukhuset Malmö, Cypressvägen 11, Box 9090, 200 39 Malmö, Sweden

**Keywords:** Bitch, Uterine inflammation, Surgical treatment, Hospitalization, Peritonitis, Risk, Outcome, Dogs

## Abstract

**Background:**

One of the most common diseases in intact bitches is pyometra– a potentially life-threatening disease associated with a variety of clinical and laboratory findings. The aims of the present study were to describe complications of the disease and to investigate clinically useful indicators associated with peritonitis and/or prolonged postoperative hospitalization.

**Results:**

A retrospective study was performed using records from 356 bitches diagnosed with pyometra during the years 2006–2007 at the University Animal Hospital, Swedish University of Agricultural Sciences, Uppsala, Sweden. Of the 356 bitches, 315 were surgically treated by ovariohysterectomy, 9 were medically treated and 32 were euthanized without treatment. In the surgically treated bitches, univariable associations between clinical and laboratory data, risk for prolonged hospitalization (≥ 3 days) and/or signs of peritonitis, were analyzed by Chi-square and Fisher’s exact test. Logistic regression models were used to assess multivariable associations.

The most common complication observed in surgically treated bitches was peritonitis (40 bitches), followed by urinary tract infection (19 bitches), wound infection (8 bitches), uveitis (6 bitches), and cardiac arrhythmia (5 bitches). Leucopenia and fever/hypothermia were associated with increased risk for peritonitis (18-fold and three-fold, respectively). Moderate to severe depression of the general condition, pale mucous membranes and leucopenia were associated with increased risk (seven-fold, three-fold, and over three-point-five-fold, respectively) for prolonged postoperative hospitalization.

**Conclusions:**

Several clinically useful indicators were identified. Leucopenia was the most important marker, associated with 18-fold increased risk for peritonitis and an over three-point-five increased risk for prolonged hospitalization. Fever/hypothermia, depression and pale mucous membranes were associated with increased risk for peritonitis and/or prolonged hospitalization. The results of the present study may be valuable for identifying peritonitis and predicting increased morbidity in surgically treated bitches with pyometra.

## Background

One of the most common diseases in intact bitches is pyometra affecting approximately 25% before 10 years of age [1]. Differences in incidence rates between breeds have been described [2-4]. The diagnosis is based on case history, physical examination, and laboratory analyses, often combined with radiography and/or ultrasonography of the uterus and ovaries. Clinical signs vary depending on severity of disease [5,6]. Leucocytosis, neutrophilia with left shift, anaemia, monocytosis, hypoalbuminemia as well as affected liver or kidney function are common findings [7]. Pyometra has deadly consequences if left untreated and despite modern treatment routines the mortality is 3-4% [1]. The safest and most effective treatment is ovariohysterectomy (OHE) but purely medical treatment can be used in selected cases [8]. Though OHE is a routine procedure, anesthesia and surgery in bitches suffering from severe systemic disease or/and organ malfunctions may be hazardous [9]. The majority of bitches with pyometra suffer from systemic inflammatory response syndrome, which previously has been associated with increased hospitalization and mortality rates [10]. It is important, but clinically difficult, to predict outcome which is why indicators for survival, complications and morbidity are wanted. Prognosis or mortality prediction by investigating different physical status and laboratory parameters is currently in demand in both human and veterinary medicine. Guidelines for performing anesthesia and assessing anesthetic risk based on different criteria are also being developed [11,12]. Most clinical variables are, however, unspecific which is why current research focuses on identifying clinically valuable biomarkers with high sensitivity and specificity. In diseases with low mortality, such as pyometra, duration of postoperative hospitalization has been used as a measure for morbidity [5,13-18]. The present study explored clinical and laboratory parameters as indicators of morbidity, measured by duration of postoperative hospitalization and/or peritonitis, using analysis of multivariable associations.

Potentially life-threatening complications of pyometra, described after surgery, include sepsis, septic shock, disseminated bacterial infection, peritonitis and hemorrhage [19-21]. Clinical signs such as vomiting, diarrhea, abdominal distention, decreased appetite or abdominal pain may be observed in animals with septic peritonitis [22,23]. These signs are, however, commonly encountered in bitches suffering from pyometra with or without peritonitis [24]. For this purpose, researchers have studied biomarkers as indicators of severity of disease and outcome [13,25]. However, adding analyses that are not routinely performed in clinical work may be time consuming and the cost benefit must be considered. The value of clinical analyses and variables routinely available such as case history data, clinical signs, physical examination findings or laboratory biomarkers has not yet been fully explored as indicators of outcome after surgical treatment of pyometra.

The aims of the present study were to describe complications of pyometra and to investigate variables that may be useful as indicators of peritonitis and/or prolonged hospitalization after surgical treatment.

## Results

During the years 2006–2007, 356 bitches of 92 different breeds were diagnosed with pyometra. The age range was one to 15 years (mean ± SD, 9 ± 1.4 years). Seventy-two bitches were > 10 years old. All bitches were diagnosed within 2 months of the previous oestrus. Oestrus prevention by medroxyprogesterone acetate (MPA, the only registered treatment in Sweden) is rare in Sweden, and was only given to one bitch in the study material.

### Case history data, physical examination and laboratory findings

Case history, physical and laboratory examination data from the 356 bitches with pyometra are shown in Table 1.

**Table 1:** Case history, physical and laboratory examination data as recorded in 356 bitches with pyometra

**Variable**	**In no of bitches/ total no of bitches with data recorded**	**Proportion of bitches with respective finding (%)**
**Case history**		
Vaginal discharge	237/309	76.7
Anorexia	193/280	69.0
Depression	225/356	63.0
Polydipsia	180/292	61.6
Polyuria	171/288	59.4
Vomiting	75/356	21.1
Lameness	56/342	16.4
Diarrhea	55/356	15.4
Urinary tract infection	19/342	5.6
**Clinical findings**		
Fever	96/301	31.9
Dehydration	94/356	26.4
Abdominal pain on palpation	81/356	22.7
Palpable enlarged uterus	67/356	18.8
Hyperemic mucous membranes	58/356	16.3
Pale mucous membranes	52/356	14.6
Hypothermia	12/301	4.0
**Laboratory analyses**		
**Hematology**		
Anemia	88/177	49.7
Neutrophilia	119/215	55.3
Leucocytosis	121/223	54.3
Monocytosis	108/213	50.7
Band neutrophils	31/208	14.9
Toxic neutrophils	21/223	9.4
Leucopenia	8/223	3.6
Neutropenia	8/215	3.7
Monocytopenia	7/213	3.3
**Clinical chemistry**		
Increased ALP	71/192	37.0
Increased bile acids	7/30	23.3
Increased lactate	2/20	10.0
Increased BUN	2/31	6.5
Increased creatinine	11/228	4.8
Hypoglycemia	7/156	4.5
Hyperglycemia	6/156	3.8

Normal = Within the reference range for healthy bitches and for laboratory variables at the Clinical chemistry laboratory, University Animal Hospital, Swedish University for Agricultural Sciences, Sweden.

Data listed includes the number of bitches/the total number of bitches with data recorded and proportion (%) with respective finding.

### Treatment alternatives

In total, 315 bitches were surgically treated by OHE. In 65 surgically treated bitches (21%), antimicrobial therapy had been administered prior to admission, and in 124 bitches (35%) antimicrobials were administered postoperatively. Nine bitches were selected for medical treatment. Thirty-two bitches (9%) were euthanized after diagnosis without treatment.

### Mortality

The total mortality was 10% (36/356) including euthanasia of 32 bitches and postoperative death of four bitches (1%) (Table 2). Of the bitches that died postoperatively, one died due to splenic rupture, one due to severe peritonitis and two of unknown causes (Table 3). Euthanasia was performed due to concomitant diseases including severe hip dysplasia (n = 1), hepatic disease associated with ascites (n = 1), long-term polyuria/polydipsia (n = 1), several other diseases (n = 2), kidney malfunction (n = 1), mammary tumors (n = 1), multiple neoplasia in esophagus (n = 1), or due to pyometra associated with old age of the bitch (n = 24). None of the bitches were euthanized because of a poor prognosis of pyometra.

**Table 2:** Total number, mortality, cases diagnosed with peritonitis or that had prolonged hospitalization (numbers and proportions) in bitches with pyometra that were euthanized, treated surgically or medically

**Bitches**	**n**	**Mortality n (%)**	**Peritonitis n (%)**	**Prolonged hospitalization n (%)**
All	356	36 (10%)	44 (12%)	60 (19%)
Euthanized	32	32 (100%)	4 (12.5%)	0 (0%)
Surgically treated	315	4 (1%)	40 (13%)	60 (19%)
Medically treated	9	0 (0%)	0 (0%)	0 (0%)

**Table 3:** Clinical signs, findings on physical and laboratory examinations and cause of death of the four bitches that died after surgical treatment of pyometra

**Bitch**	**Case history**	**Physical status**^**a**^	**Laboratory analyses**^**b**^	**Cause of death**
No. 1	Polyuria, polydipsia, bloody vaginal discharge, mild depression	Hypothermia, mild dehydration, CRT 1–2 sec.	Leucocytosis	Unknown
No. 2	Anorexia, severe depression	Severe dehydration, abdominal pain, hyperemic mucous membrane, CRT > 2 sec.		Severe peritonitis
No. 3	Anorexia, bloody-purulent vaginal discharge, mild depression	CRT 1–2 sec.	Anemia, increased ALP, increased lactate	Ruptured spleen
No. 4	Vomiting, diarrhea, purulent vaginal discharge, severe depression		Leucopenia, increased ALP	Unknown

^a^CRT = Capillary refill time; ^b^ALP = Alkaline phosphatase.

### Complications in all pyometra patients

Complications reported in the 356 bitches with pyometra were peritonitis (12.4%, n = 44), urinary tract infection (5.3%, n = 19), wound infection (2.2%, n = 8), uveitis (1.7%, n = 6), cardiac arrhythmia (1.4%, n = 5), persistent polyuria/polydipsia (0.3%, n = 1), hepatic disease associated with ascites (0.3%, n = 1) and kidney malfunction (0.3%, n = 1).

### Complications in surgically treated pyometra cases

In total, there were specific complications and prolonged postoperative hospitalization of the surgically treated bitches observed in 25% (78/315) and 19% (60/315), respectively. The specific complications observed were peritonitis (13%, 40/315) including eight bitches with ruptured uterus, urinary tract infection (6%, 19/315), wound infection (3%, 8/315), uveitis (2%, 6/315), and cardiac arrhythmias (1%, 5/315).

### Indicators for prolonged postoperative hospitalization and/or peritonitis

Clinical signs, physical examination findings and laboratory variables that were investigated for possible associations with prolonged hospitalization (≥ 3 days) or peritonitis are shown in Tables 4 and 5, respectively. These analyses were only performed in surgically treated bitches. The age and weight did not differ significantly between bitches with or without peritonitis or prolonged hospitalization (data not shown). Other variables not shown were not associated with peritonitis and/or prolonged hospitalization. Results of the multivariable analyses are presented in Tables 6 and 7. The Hosmer-Lemeshow goodness-of-fit statistics were not significant (p = 0.86 and p = 0.71, respectively) indicating a good fit of the multivariable models. The models explained 30 and 21% of the variation, as assessed by the generalized R^2^.

**Table 4:** Univariable analysis of association between clinical signs, physical examination findings and laboratory data and the risk of prolonged postoperative hospitalization (≥ 3 days) in bitches with pyometra

**Variable**^**a**^		**No. of bitches with prolonged hospitalization/bitches with variable n (%)**	**Missing data**	**p-value (Chi-square test/Fisher’s exact test)**
**Case history**				
Anorexia	Yes	34/167 (20)	69	0.08
	No	9/79 (11)		
Polyuria	Yes	25/151 (16)	62	0.17
	No	24/102 (23)		
Polydipsia	Yes	24/159 (15)	59	0.04
	No	25/97 (16)		
Vomiting	Yes	21/68 (31)	0	0.005
	No	39/247 (16)		
Diarrhea	Yes	13/51 (25)	0	0.2
	No	47/264 (18)		
Vaginal discharge	Yes	44/205 (21)	42	0.5
	No	12/68 (18)		
Depression	Normal (brightness)	12/126 (9)	0	< 0.0001
	Mild	20/116 (17)		
	Moderate	15/54 (28)		
	Severe	13/19 (68)		
Lameness	Yes	13/48 (27)	14	0.11
	No	44/253 (17)		
Urinary tract infection	Yes	3/19 (16)	14	0.7
	No	54/282 (19)		
**Physical examination**				
Body temperature	Normal	31/175 (18)	50	0.4
	Fever	20/82 (24)		
	Hypothermia	1/8 (12)		
CRT	Normal	25/133 (19)	165	0.11
	Abnormal	6/17 (35)		
Mucous membranes	Normal	35/220 (16)	0	0.01
	Pale	14/43 (33)		
	Hyperemic	10/51 (20)		
	Toxic	1/1 (100)		
Hydration status	Normal	37/232 (16)	0	0.004
	Mild	15/67 (22)		
	Moderate	7/15 (47)		
	Severe	1/1 (100)		
Abdominal pain	Yes	20/69 (29)	0	0.02
	No	40/246 (16)		
Palpable uterus	Yes	13/59 (22)	0	0.5
	No	47/256 (18)		
Ophthalmological exam	Ocular discharge	56/287 (19)	0	0.48
	Conjunctivitis	4/22 (18)		
	Uveitis	0/6 (0)		
**Laboratory analyses**				
WBC	Normal	21/90 (23)	102	< 0.0001
	Leucocytopenia	7/8 (87)		
	Leucocytosis	16/115 (14)		
Neutrophils	Normal	19/85 (22)	110	< 0.0001
	Neutropenia	7/8 (87)		
	Neutrophilia	15/112 (13)		
Band neutrophils	Normal	33/166 (20)	118	0.25
	Increased	9/31 (29)		
Toxic neutrophils	Yes	7/21 (33)	102	0.1
	No	37/192 (19)		
Monocytes	Normal	23/90 (26)	112	0.0004
	Monocytopenia	5/7 (71)		
	Monocytosis	14/106 (13)		
Hb	Normal	9/32 (28)	260	0.7
	Low	5/21 (24)		
	High	1/2 (50)		
Hct	Normal	15/85 (18)	148	0.38
	Anemia	19/82 (23)		
ALP	Normal	17/113 (15)	134	0.48
	Decreased	1/4 (25)		
	Increased	14/64 (22)		
Creatinine	Normal	35/209 (17)	100	0.0009
	Increased	4/6 (67)		
Bile acids	Normal	4/23 (17)	285	0.5
	Increased	2/7 (29)		
BUN	Normal	5/26 (19)	288	0.09
	Uremia	1/1 (100)		
Glucose	Normal	24/137 (17)	167	0.01
	Hypoglycemia	4/6 (67)		
	Hyperglycemia	1/5 (20)		
Lactate	Normal	4/17 (23)	296	0.03
	Increased	2/2 (100)		

^a^Normal = Within the reference range (as indicated below) for healthy bitches and for laboratory variables at the main Clinical chemistry laboratory, University Animal Hospital, Swedish University for Agricultural Sciences, Sweden. CRT = Capillary refill time (1–2 s), WBC = White Blood Cell Count (5.8-16.0 ×10^9^/L), Hb = Hemoglobin (132–199 g/L), Hct = Hematocrit (38-57%), ALP = Alkaline phosphatase (<5.0 μkat/L), BUN = Blood urea nitrogen (2.5-8.5 mmol/L), Neutrophils (3.0-11.5 ×10^9^/L), Monocytes (0.2-1.4 x10^9^/L), Creatinine (40–130 μmol/L), Bile acids (<10 μmol/L), Glucose (4.5-5.8 mmol/L), Lactate levels (<2.2 mmol/L), Body temperature (38–39.2°C).

**Table 5:** Univariable analysis of associations between clinical signs, physical examination findings and laboratory data and presence of peritonitis in bitches with pyometra

**Variable**^**a**^		**No of bitches with peritonitis/ bitches with variable n (%)**	**Missing data**	**p-value (Chi-square test/Fisher’s exact test)**
**Case history**				
Anorexia	Yes	22/166 (13)	70	0.05
	No	4/79 (5)		
Polyuria	Yes	17/148 (11)	65	0.34
	No	16/102 (16)		
Polydipsia	Yes	15/154 (10)	64	0.07
	No	17/97 (17)		
Vomiting	Yes	14/64 (22)	8	0.01
	No	25/243 (10)		
Diarrhea	Yes	8/51 (16)	8	0.5
	No	31/256 (12)		
Vaginal discharge	Yes	27/203 (13)	44	0.99
	No	9/68 (13)		
Depression	Normal (brightness)	8/119 (7)	8	< 0.0001
	Mild	12/115 (10)		
	Moderate	11/54 (20)		
	Severe	8/19 (42)		
Lameness	Yes	8/48 (17)	14	0.4
	No	31/253 (12)		
Urinary tract infection	Yes	1/19 (5)	14	0.3
	No	38/282 (13)		
**Physical examination**				
Body temperature	Normal	16/170 (9)	57	0.006
	Fever	19/81 (23)		
	Hypothermia	0/7 (0)		
CRT	Normal	15/133 (11)	166	0.03
	Abnormal	5/16 (31)		
Mucous membranes	Normal	22/213 (10)	8	0.02
	Pale	8/42 (19)		
	Hyperemic	8/51 (16)		
Hydration status	Normal	25/224 (11)	8	0.004
	Mild	8/67 (12)		
	Moderate	5/15 (33)		
	Severe	1/1 (100)		
Abdominal pain	Yes	14/67 (21)	8	0.02
	No	25/240 (10)		
Palpable uterus	Yes	6/58 (10)	8	0.5
	No	33/249 (18)		
Ophthalmological exam	Ocular discharge	37/279 (13)	8	0.5
	Conjunctivitis	2/22 (9)		
	Uveitis	0/6 (0)		
**Laboratory analyses**				
WBC	Normal	10/90 (11)	102	< 0.0001
	Leucopenia	3/5 (62)		
	Leucocytosis	11/115 (10)		
Neutrophils	Normal	9/85 (11)	110	< 0.0001
	Neutropenia	3/5 (62)		
	Neutrophilia	10/112 (9)		
Band neutrophils	Yes	18/166 (11)	118	0.07
	Increased	7/31 (23)		
Toxic neutrophils	Yes	4/21 (19)	102	0.3
	No	22/192 (11)		
Monocytes	Normal	12/90 (13)	112	0.02
	Monocytopenia	3/7 (43)		
	Monocytosis	9/106 (8)		
Hb	Normal	5/32 (16)	260	0.5
	Dereased	4/21 (19)		
	Increased	1/2 (50)		
Hct	Normal	10/85 (12)	148	0.9
	Anemia	9/82 (11)		
ALP	Normal	14/108 (13)	141	0.48
	Decreased	0/4 (0)		
	Increased	5/62 (8)		
Creatinine	Normal	21/202 (10)	107	0.01
	Increased	1/5 (20)		
Bile acid	Normal	3/23 (13)	285	0.9
	Increased	1/7 (14)		
BUN	Normal	5/26 (19)	285	0.63
	Uremia	0/1 (0)		
Glucose	Normal	16/133 (12)	173	0.05
	Hypogycemia	2/4 (50)		
	Hyperglycemia	0/5 (0)		
Lactate	Normal	4/17 (23)	296	0.4
	Increased	1/2 (50)		

^a^Normal = Within the reference range (as indicated below) for healthy bitches and for laboratory variables at the Clinical chemistry laboratory, University Animal Hospital, Swedish University for Agricultural Sciences, Sweden. CRT = Capillary refill time (1–2 s), WBC = White Blood Cell Count (5.8-16.0 ×10^9^/L), Hb = Hemoglobin (132–199 g/L), Hct = Hematocrit (38-57%), ALP = Alkaline phosphatase (<5 μkat/L), BUN = Blood urea nitrogen (2.5-8.5 mmol/L), Neutrophils (3.0-11.5 ×10^9^/L), Monocytes (0.2-1.4 ×10^9^/L), Creatinine (40–130 μmol/L), Bile acids (<10 μmol/L), Glucose (4.5-5.8 mmol/L, ), Lactate levels (<2.2 mmol/L), Body temperature (38–39°C).

**Table 6:** Multivariable logistic regression model of association between clinical signs, physical examination findings and laboratory data and the risk of prolonged postoperative hospitalization (≥ 3 days) in bitches with pyometra (n = 184)

**Variable**^**a**^		**Estimated coefficient**	**Odds Ratio (95% confidence interval)**	**p-value**
Polydipsia^b^	Yes	-0.83 (±0.44)	0.43	0.056
			(0.18 - 1.02)	
Vomiting^b^	Yes	0.77 (±0.46)	2.16	0.097
			(0.87 - 5.38)	
Depression	Mild	1.14 (±0.57)	3.14	0.008
			(1.02 - 9.64)	
	Moderate to severe	1.91 (±0.61)	6.78	
			(2.03 - 22.59)	
Mucous membranes	Pale	1.13 (±1.37)	3.09	0.021
			(1.06 - 8.96)	
	Hyperemic	-0.95 (±0.64)	0.39	
			(0.11 - 1.35)	
WBC	Leucopenia	1.26 (±1.37)	3.53	0.012
			(0.24 - 51.73)	
	Leucocytosis	-1.31 (±0.49)	0.27	
			(0.1 - 0.71)	

^a^Normal = Within the reference range for healthy bitches and for laboratory variables at the main Clinical chemistry laboratory, University Animal Hospital, Swedish University for Agricultural Sciences, Sweden; WBC = White blood cell count (ref. range 5.8-16 ×10^9^/L); ^b^The variable is not statistically significant (p > 0.05), but included as a confounder in the model.

**Table 7:** Multivariable logistic regression model of associations between clinical signs, physical examination findings and laboratory data and the presence of peritonitis in bitches with pyometra (n = 158)

**Variable**^**a**^		**Estimated coefficient**	**Odds Ratio (95% confidence interval)**	**p-value**
Polydipsia^b^	Yes	-0.91 (±0.53)	0.40	0.084
			(0.14 - 1.13)	
Body temperature	Fever/hypothermia	1.19 (±0.50)	3.30	0.017
			(1.23 - 8.82)	
WBC	Leucopenia	2.90 (±1.20)	18.11	0.043
			(1.74 - 188.92)	
	Leucocytosis	-0.10 (±0.54)	0.90	
			(0.32 - 2.56)	

^a^Normal = Within the reference range for healthy bitches and for laboratory variables at the main Clinical chemistry laboratory, University Animal Hospital, Swedish University for Agricultural Sciences, Sweden; WBC = White blood cell count (ref. range 5.8-16 ×10^9^/L); Body temperature (ref. range 38-39°C); ^b^The variable is not statistically significant (p > 0.05), but included as a confounder in the model.

## Discussion

Identifying complications in bitches with pyometra is vital for selecting optimal monitoring routines and treatments and for determination of prognosis. In the present study, complications were observed in 25% of the bitches treated by OHE. Peritonitis was the most common complication and can be life-threatening [26]. Urinary tract infection (UTI) was the second most common complication (6%). Previously, subclinical UTI with the same bacterial strain as in the uterus has been demonstrated in 25% of bitches with pyometra [27]. Though subclinical UTI may resolve without intervention after OHE, proteinuria or clinical signs of disease should be monitored to prevent severe renal disease from developing [28]. In the present study, uveitis was diagnosed in six bitches. Uveitis has not previously been associated with pyometra but has been described in dogs and cats suffering from severe bacterial infection [29,30]. Cardiac arrhythmia, as identified in five bitches, could have been induced by endotoxemia or myocardial injury [21,31]. Peritonitis, uveitis and cardiac arrythmias are serious, but treatable, consequences of endotoxemia and sepsis, further supporting the importance for predicting such complications early [32-34].

In the present study, the total mortality including euthanasia was 10% which is higher than earlier reported [1]. Our previous study included bitches < 10 years whereas the present study incorporated data from bitches of all ages, hence the 20% that were > 10 years may contribute to some extent to explain this difference since the owners could be more likely to choose elective euthanasia instead of treatment in an older dog or that has concurrent diseases (which is more likely in older dogs). Four bitches died postoperatively, resulting in a mortality of 1% after surgery, which is lower than the 5-27% in previous reports [19,31,35]. Surgical treatment of pyometra had thus a very good prognosis, when performed in the selected cases, in our study. Thirty-two (9%) bitches were euthanized instead of treated due to old age and/or concurrent diseases (such as mammary tumor), all by request of the owner and in agreement with the veterinary surgeon in charge. These bitches were excluded from the analysis of predictors for increased postoperative hospitalization, peritonitis and mortality because they were not surgically treated and their death was not associated with a poor prognosis or severity of pyometra. Age and weight did not differ between the groups (without complications and hospitalization < 3 days compared to with complications and/or prolonged hospitalization), showing that peritonitis or prolonged hospitalization was not more common in older bitches. This indicates that age by itself is not a risk-factor for surgical treatment of pyometra [9].

Overall, the most common signs of disease, present in > 50% of the bitches, included vaginal discharge, anorexia, depression, polydipsia, and polyuria (Table 1), reflecting systemic involvement of the disease in the majority of bitches [19,36,37]. Vaginal discharge, which has been associated with more severe disease and is considered a characteristic sign of pyometra [6], was absent in 23% of the cases. Obscure signs of illness may make the disease more difficult to recognize and supports the need for diagnostic indicators for pyometra. Interestingly, lameness was present in 56 (16%) of the bitches, which has not been reported previously. It is possible that the activated immune response could, for example, trigger arthritis, as has been suggested in humans [38]. Regarding laboratory findings, leucocytosis with neutrophilia and left shift, monocytosis and anaemia were observed in the majority of bitches, as is common in the disease [19,39].

One of 40 bitches with peritonitis died, resulting in a 3% mortality in pyometra with peritonitis which is comparatively low compared with mortality rates of 50% previously reported due to uterine rupture [26]. Importantly, none of the bitches with ruptured uterus died. We may speculate that this could depend on the disease being common in Sweden and possible complications recognized earlier compared with in countries where the majority of the bitches are neutered.

All surgically treated bitches were included in the analyses for identifying indicators for peritonitis and/or prolonged postoperative hospitalization. Two bitches were diagnosed with peritonitis postoperatively and since it could have been subclinical at the time of surgery or caused by uterine leakage during surgery, these bitches were included in the peritonitis group when analyzing for indicators. Moderate to severe depression and pale mucous membranes were associated with increased risk of prolonged postoperative hospitalization (Table 6). A striking result was that leucopenia was associated with a three-point-five-fold increased risk of having prolonged hospitalization compared to normal WBC and also an 18-fold increased risk of peritonitis. These results make leucopenia the most important clinical biomarker identified. Leucopenia could be caused by endotoxin-induced bone marrow depression in combination with more chronic inflammatory disease and loss of leucocytes to the uterine lumen. Increased mortality has also been demonstrated in animals and humans with leucopenia in other studies [40-42]. A WBC within the normal reference range was associated with increased risk for prolonged hospitalization and/or peritonitis as compared to leucocytosis. This could possibly reflect a transition from leucocytosis to leucopenia i.e. leucocytosis appearing earlier in the pathogenesis. Not only may the number of leucocytes be decreased in pyometra, but their function (phagocytic capacity and mitogen-driven lymphocyte proliferation) is also impaired, negatively affecting the combat against infection [43,44]. Bitches with moderately to severely depressed general condition had a seven-fold increased risk for prolonged hospitalization. Pale mucous membranes, which might reflect anemia, were associated with a three-fold increased risk of prolonged hospitalization. In contrast, hyperemic mucous membranes were associated with decreased risk for prolonged hospitalization. Lactate levels, though only analyzed in 19 dogs, were associated with increased risk for prolonged hospitalization, indicating a predictive value.

Other variables than leucopenia were linked with presence of peritonitis. Fever, or hypothermia present in merely a third of the pyometra cases, was associated with a three-fold increased risk of peritonitis indicating a prognostic value for this variable. Uterine diameter was not associated with peritonitis or prolonged hospitalization, which would otherwise be plausible since a larger uterus could have indicated more severe local disease.

Some parameters were analyzed only in a few bitches hence missing data is a limitation in our study. Retrospectively collected data may also be less reliable than prospectively collected data. Because the bitches selected for medical treatment were already less severely affected by their disease than those subjected to surgical treatment, the data reported here cannot serve as a comparison of the two treatment methods. The results of the present study will increase the possibilities to predict prognosis and outcome after surgical treatment of pyometra. This will be valuable in early identification of cases with peritonitis and/or increased morbidity (hospitalization), which will in turn aid in treatment selection and thereby possibly also increase survival.

## Conclusions

Complications such as peritonitis, uveitis, urinary tract infection, wound infection and cardiac arrhythmias were observed in bitches with pyometra. Several routine parameters that may be useful as indicators of peritonitis and/or prolonged hospitalization were identified. Leucopenia was associated with increased risk for peritonitis and prolonged hospitalization making leucopenia the most important biomarker to be aware of clinically. The results of the present study will be clinically valuable for identifying peritonitis in bitches with pyometra and the prediction of prolonged postoperative hospitalization after surgical treatment.

## Methods

### Animals

Only journal data already available was used for the study and ethical approval therefore not necessary to obtain according to Swedish regulations.

A retrospective study was carried out using data records from all bitches diagnosed with pyometra during the years 2006–2007 at the University Animal Hospital (UDS), Swedish University of Agricultural Sciences (SLU), Uppsala, Sweden. Bitches were identified by the diagnostic code for pyometra used in Sweden [26]. The animal hospital’s patient records include data such as breed, weight, age, case history, physical examination findings, results of radiographic and/or ultrasonographic examinations, laboratory analyses including hematology and serum biochemistry, treatments, date of dismission and follow-ups at the UDS. The preliminary diagnosis pyometra was based on the results of case history, physical examination and diagnostic imaging. The cases were admitted mainly within 2 months of previous oestrus (in metoestrus), not associated to parturition or pregnancy and all dogs had signs of systemic illness. Ultrasonography or radiography or both were used to demonstrate an enlarged, fluid-filled uterus. The diagnosis pyometra was verified visually during ovariohysterectomy and according to the previous definitions by De Bosschere and others (2001) [45]. Bitches with cystic endometrial hyperplasia, mucometra, hydrometra, hematometra and endometritis were not included. The bitches diagnosed with pyometra were divided into three groups depending on whether they were euthanized, medically or surgically treated. Euthanasia was performed at the request of the owner and in agreement with the veterinary surgeon in charge due to concomitant diseases. Bitches with normal hydration status, unaffected or slightly depressed general condition and with no ovarian or endometrial cysts demonstrated on ultrasonographic examination were selected for medical treatment with aglepristone (Alizin vet®, Virbac, France) in combination with antimicrobials. The success of the medical treatment was evaluated by ultrasonography and laboratory tests including hematology, total white blood cell counts and differential counts to monitor the treatment response. In this study, all medically treated cases recovered as judged by normal laboratory tests and no uterine or ovarian pathology on diagnostic imaging. Furthermore, data from the surgically treated bitches were analyzed for indicators of preexisting peritonitis or development of postoperative peritonitis or prolonged postoperative hospitalization. Intraocular pressure was measured in all bitches with uveitis.

### Variables as indicators of peritonitis and/or prolonged hospitalization

Variables included in the analyses for indicators of peritonitis and/or prolonged postoperative hospitalization were as follows: appetite, body temperature, depression, mucous membrane appearance, hydration status, capillary refill time (CRT), polyuria, polydipsia, vomiting, diarrhea, vaginal discharge, lameness, urinary tract infection, presence of abdominal pain on palpation, palpable enlarged uterus and all other pathological findings noted when performing a complete physical examination. The following laboratory variables were included in the analyses: total white blood cell count (WBC) with differential counts and morphology, hemoglobin (Hb), hematocrit (Hct), alkaline phosphatase (ALP), creatinine, bile acids, blood urea nitrogen (BUN), glucose and lactate concentrations. Additionally, antimicrobial administration, ligation material used during surgery, presence of peritonitis at surgery, administered drugs (anesthetic agents, analgesics, i.v. fluid therapy), duration of postoperative hospitalization and uterine diameter (as determined by ultrasonography or macroscopically during surgery) were integrated in the analyses. The study defined abnormality of hematology and blood chemistry by using the reference ranges at the main clinical chemistry laboratory, UDS, SLU. Case history, laboratory and clinical examination variables recorded before surgery were used in the analysis.

### Determination of prolonged hospitalization and peritonitis

In general, bitches subjected to OHE due to pyometra at UDS are hospitalized 1–2 days. Prolonged postoperative hospitalization (defined as ≥ 3 days) is only warranted if specific complications occur, if the general condition is depressed and the bitch requires additional veterinary care and monitoring (considered as an unspecific complication). Peritonitis was identified by free fluid and/or hyperechoic fat tissue (steatitis) detected on ultrasonographic examination of the abdomen or decreased serosal detail observed on radiographic examination (as a consequence of steatitis and/or intra-abdominal fluid). Macroscopically peritonitis was identified visually by fibrin or other signs of inflammation on the surface of the abdominal structures or pus present in the abdominal cavity or by positive bacterial culture from the abdominal fluid. In two bitches, peritonitis was diagnosed the day after surgery.

### Statistical analysis

Univariable associations between potential risk factors within case history, physical examination and laboratory data and the outcomes prolonged hospitalization and signs of peritonitis, respectively, were analyzed by Chi-Square test and Fisher’s exact test. Multivariable associations between these potential risk factors and the outcomes were analyzed by logistic regression models. All variables with a p-value ≤ 0.20 in the univariable analyses were considered as potential predictor variables. Categorical predictor variables were introduced in the models coded as dummy variables. Collinearity between potential predictor variables were assessed by variance inflation factors (VIF) above 10 [46] in which case the variable with a) least missing values or b) providing the best model fit was retained. Modeling was done manually by backward elimination of non-significant (p > 0.05) variables. At each step, previously eliminated variables were tested for reentry. Confounding was assessed by comparing the change in estimated coefficients when variables were excluded from the model, and were considered present if a coefficient changed > 20%. The fit of the final multivariable model was assessed with a Hosmer-Lemeshow goodness-of-fit test [47] and the coefficient of determination was assessed with a generalized *R*^2^ as suggested by Nagelkerke (1991) [48]. All statistical analyses were performed using SAS (version 9.3, SAS Institute Inc., Cary, NC, USA).

## Competing interests

None of the authors have any conflict of interest to declare.

## Authors’ contributions

SJ drafted the manuscript. CAB, SJ, RH, AP, and OH provided data and managed the data records. UE performed statistical analyses. RH, AP, BSH, CAB, UE and OH reviewed and commented the manuscript during its preparation. All authors read and approved the final manuscript.
